# Campbell Title Registrations to Date – February 2025, and Discontinued Protocols

**DOI:** 10.1002/cl2.70034

**Published:** 2025-03-10

Details of new titles for systematic reviews or evidence and gap maps that have been accepted by the Editor of a Campbell Coordinating Group are published in each issue of the journal. If you would like to receive a copy of the approved title registration form, please *send an email* to the Managing Editor of the relevant Coordinating Group.

A list of discontinued protocols appears below these new titles. If you are interested in continuing a project, please get in touch with the Managing Editor of the relevant Coordinating Group or email info@campbellcollaboration.org.

## 1 Ageing

Experiences of informal caregivers of dependent older people on interventions to reduce care burden and promote their biopsychosocial well‐being

Mariana Loezar‐Hernández, Alejandra Ximena Araya, Claudia Miranda‐Castillo, Carolina Climent‐Sanz

11 February 2025

Interventions for age‐friendly environments: A mega‐map

Thiago Sa, Amanda Fernandes, Louise Lafortune, Promise Nduku

11 February 2025

## 2 Business & Management

Do pay transparency policies advance pay equity goals? A systematic review

Constance Bygrave, Heidi Weigand, Wendy Carroll, Alison Manley

31 January 2025

The impact of abusive supervision on employee silence: A systematic review

Wendy Carroll, Camilla Holmvall, Alison Manley

24 January 2025

## 3 Children and Young Persons Wellbeing

Characteristics of sustainable vocational placement youth not in employment, education, or training (NEET): A scoping review

Luther Monareng, Nomusa F. Mngoma

20 January 2025

Thresholds for placing children in foster care: A scoping review

Nella Hrda, Milon Potmesil, Petra Potmesilova

29 January 2025

## 4 Crime and Justice

Primary interventions for cognitive and behavioral radicalization: An evidence and gap map

Kieren Aris, Meriem Rebbani, Joshua Freilich

6 January 2025

Radicalisation and extremism amongst adolescents and adults: A systematic review of measurement properties

Sarah Carthy, Graig Klein, Tommy van Steen, Joana Cook

8 December 2024

## 5 Disability

Peoples' lived experiences of working with osteoarthritis: A qualitative meta‐synthesis protocol

Rory Christopherson, Ricky Bell, Nicola Kayes, J. Haxby Abbott

6 February 2025

## 6 Education

Mitigating the risk of large language models on academic integrity of higher education assessment: A systematic review

David Oyebisi

19 December 2024

## 7 International Development

The effects of land management policies on the environment and people in low‐ and middle‐income countries: A systematic review

Pierre Marion, Ingunn Storhaug, Sanghwa Lee, Claudia Romero, Constanza Gonzalez, Birte Snilstveit

20 December 2025

Life skills education and psychosocial interventions for reducing anxiety and depression in forcibly displaced persons in LMICs: A systematic review

Andem Duke

12 February 2025

Interventions to prevent or reduce the effects of climate change on the well‐being of socially vulnerable populations in low‐ and middle‐income countries: An evidence and gap map

Alfonso Apicella, Sandy Oliver, Janice Tripney

12 February 2025

## 8 Knowledge Translation and Implementation

Barriers and facilitators to the adoption of speech‐based cognitive screening tools: A protocol for systematic review of qualitative evidence

Ravi Shankar, Anjali Bundele, Amartya Mukhopadhyay

13 January 2025

Paving the way for improved healthcare: A realist review protocol to identify contextual factors and mechanisms that facilitate or hinder cardiovascular self‐management and recovery behaviours in adults with severe mental illness

Sara Zabeen, Lenore Perrelle, Leila Mohammadi, Sharon Lawn, Anthony Venning, Kate Fairweather, Sonia Hines

13 February 2025

Protocol of scoping review to map evidence and identify gap on the mitochondrial function and bioenergetics following physical exercise

Muhammad Hafiz Zuhdi Fairof, Arimi Fitri Mat Ludin, Muhammad Fiqry Mohd Aris, Norwahidah Abd Karim, Nurul Hidayah Md Fadzil, Fatin Hanani Mazri, Nor Fadilah Rajab

13 February 2025

What are the effective strategies to increase the involvement of women in leadership and decision‐making positions in public institutions in LMICs?

Fasil Deribe, Prisca Auma, Desalegn Garoma/Ararso/Desalegn, Joel Acana, Nebiyou Gebreyesus

13 January 2025

## 9 Social Welfare

Psychosocial interventions for improving mental health outcomes in healthcare workers employed in hospital settings: A systematic review and meta‐analysis

Harly Blumhagen, Brandy Maynard

17 February 2025

Effectiveness of virtual reality in supporting informal caregivers: A protocol for a systematic review and meta‐analysis

Ravi Shankar, Fiona Devi, Amartya Mukhopadhyay

10 February 2025

Global willingness to pay for COVID‐19 vaccines: Protocol for a systematic review and meta‐analysis

Nizam Abdu

24 January 2025

Health care provider's experiences, attitudes, values, and beliefs, and how these relate to improving the experiences of the sexual and gender diverse when they seek care: A qualitative systematic review

Tyler Glass, Marjan Kekanovich, Amanda Ross‐White, Marian Luctkar‐Flude, Roger Pilon, Jacqueline Galica

24 January 2025

Effectiveness of intimate partner violence versus absence of intimate partner violence in pregnant women worldwide: A systematic review

Adriano Belarmino, Patrícia Bastos, Antonio Ferreira Junior, José Carvalho Junior

24 January 2025

Healthcare professionals' knowledge, attitudes, and practices in providing mental health care for queer individuals: An evidence and gap map of observational and intervention studies

Martin Gossi, Dylan Kneale

10 December 2024

Community‐based interventions to improve the health of those experiencing houselessness in high‐income countries: An evidence and gap map

April Ballard, Shannon Self‐Brown

24 January 2025

A systematic review of global research studies on community health workers in pharmacy settings to advance the implementation of behavioral health

Emily Palm, Phillip Marotta, Heather Lyons‐Burney, Amy Tiemeier, Annie Eisenbeis, Carson Von Alst

24 January 2025

## 10 Discontinued Protocols

If you are interested in continuing one of the projects below, please get in touch with the Managing Editor of the relevant Coordinating Group or email info@campbellcollaboration.org.

## 11 Business and Management

Outcomes of workplace coaching for individuals and organizations

Niklas Frewel, Rossella Barilli, Fabio Massei, Lorenzo Avanzi

Psychological empowerment and performance in the workplace

Rossella Barilli, Niklas Frewel, Fabio Massei, Jonny Gifford

Ethnic diversity and team performance

Barbara Janssen, Eric Barends

Predictors of virtual team outcomes

Iulia Ciocca, Oana Fodor, Shannon Marlow

## 12 Crime and Justice

https://onlinelibrary.wiley.com/doi/10.1002/CL2.85

PROTOCOL: Mandatory arrest for misdemeanor domestic violence effects on repeat offending

Barak Ariel, Lawrence W. Sherman

## 13 Education

https://onlinelibrary.wiley.com/doi/10.1002/CL2.215

Protocol for a systematic review: Interventions to improve mathematics achievement in primary school‐aged children: A systematic review

Victoria Simms, Camilla Gilmore, Seaneen Sloan, Clare McKeaveney

https://onlinelibrary.wiley.com/doi/10.1002/cl2.1011

PROTOCOL: Protocol for a systematic review: Inter‐school collaborations for improving educational and social outcomes for children and young people: A systematic review

Paul Connolly, Jennifer Hanratty, Joanne Hughes, Christopher Chapman, Danielle Blaylock

https://onlinelibrary.wiley.com/doi/10.1002/CL2.145

PROTOCOL: Practices and program components for enhancing prosocial behavior in children and youth: A systematic review

Asha L. Spivak, Mark W. Lipsey, Dale C. Farran, Joshua R. Polanin

https://onlinelibrary.wiley.com/doi/10.1002/CL2.185

PROTOCOL: The direct and indirect effects of school‐based executive function interventions on children and adolescents' executive function, academic, social‐emotional, and behavioral outcomes: A systematic review

Saiying Steenebergen‐Hu, Paula Olszewski‐Kubilius, Eric Calvert

https://onlinelibrary.wiley.com/doi/10.1002/CL2.163

PROTOCOL: Provision of information and communications technology (ICT) for improving academic achievement and school engagement in students aged 4–18

Kristin Liabo, Laurenz Langer, Antonia Simon, Kathy‐Ann Daniel‐Gittens, Alex Elwick, Janice Tripney

https://onlinelibrary.wiley.com/doi/10.1002/CL2.210

PROTOCOL: Universal school‐based programmes for improving social and emotional outcomes in children aged 3–11 years

Paul Connolly, Sarah Miller, Jennifer Hanratty, Jennifer Roberts, Seaneen Sloan

https://onlinelibrary.wiley.com/doi/10.1002/CL2.110

PROTOCOL: Education support services for improving school engagement and academic performance of children and adolescents with a chronic health condition

Michelle A. Tollit, Susan M. Sawyer, Savithiri Ratnapalan, Tony Barnett

https://onlinelibrary.wiley.com/doi/10.1002/CL2.197

PROTOCOL: Electronic mentoring to promote positive youth outcomes for young people under 25: A systematic review

Robyn M. O'Connor, David L. DuBois, Lucy Bowes

https://onlinelibrary.wiley.com/doi/10.1002/CL2.166

PROTOCOL: Merit pay programs for improving teacher retention, teacher satisfaction, and student achievement in primary and secondary education: A systematic review

Gary Ritter, Julie Trivitt, Leesa Foreman, Corey DeAngelis, George Denny

## 14 International Development

https://onlinelibrary.wiley.com/doi/10.1002/cl2.1122

PROTOCOL: Development evaluations in India 2000–2018: A country impact evaluation map

Ashrita Saran, Eti Rajwar, Bhumika T. V., Divya S. Patil, Howard White

https://onlinelibrary.wiley.com/doi/10.1002/cl2.1186

PROTOCOL: The association between whole‐grain dietary intake and noncommunicable diseases: A systematic review and meta‐analysis

Wasim A. Iqbal, Gavin B. Stewart, Abigail Smith, Linda Errington, Chris J. Seal

https://onlinelibrary.wiley.com/doi/10.1002/CL2.84

PROTOCOL: Nutrition interventions and programs for reducing mortality and morbidity in pregnant and lactating women and women of reproductive age: A systematic review

Philippa Middleton, Caroline Crowther, Tanya Bubner, Vicki Flenady, Zulfiqar Bhutta, Tran Son Trach, Zohra Lassi

https://onlinelibrary.wiley.com/doi/10.1002/CL2.138

PROTOCOL: Improving maternal, newborn and women's reproductive health in crisis settings

Primus Che Chi, Henrik Urdal, Odidika U. J. Umeora, Johanne Sundby, Paul Spiegel, Declan Devane

https://onlinelibrary.wiley.com/doi/10.1002/CL2.183

PROTOCOL: The impacts of interventions for female economic empowerment at the community level on human development

Marcela Ibanez, Sarah Khan, Anna Minasyan, Soham Sahoo, Pooja Balasubramanian

https://onlinelibrary.wiley.com/doi/10.1002/CL2.154

PROTOCOL: Peer‐based interventions for reducing morbidity and mortality in HIV‐positive women

J. Petkovic, M. Doull, A. M. O'Connor, J. Aweya, M. Yoganathan, V. Welch, G. A. Wells, P. Tugwell

https://onlinelibrary.wiley.com/doi/10.1002/CL2.188

PROTOCOL: Community‐led total sanitation in rural areas of low‐ and middle‐income countries: A systematic review of evidence on effects and influencing factors

Josef Novotný, Jiří Hasman, Martin Lepič, Vít Bořil

https://onlinelibrary.wiley.com/doi/10.1002/CL2.140

PROTOCOL: Access to electricity for improving health, education and welfare in low‐ and middle‐income countries

Kavita Mathur, Sandy Oliver, Janice Tripney

https://onlinelibrary.wiley.com/doi/10.1002/CL2.157

PROTOCOL: Saving promotion interventions for improving saving behaviour and reducing poverty in low‐ and middle‐income countries

Janina Isabel Steinert, Ani Movsisyan, Juliane Zenker, Ute Filipiak, Yulia Shenderovich

https://onlinelibrary.wiley.com/doi/10.1002/CL2.172

PROTOCOL: The effect of interventions for women's empowerment on children's health and education

Sebastian Vollmer, Sarah Khan, Le Thi Ngoc Tu, Atika Pasha, Soham Sahoo

https://onlinelibrary.wiley.com/doi/10.1002/CL2.200

PROTOCOL: The effects of road infrastructure, and transport and logistics services interventions on women's participation in informal and formal labour markets in low‐ and middle‐income countries

Manisha Gupta, Souvik Bandyopadhyay, Meerambika Mahapatro, Shreya Jha

https://onlinelibrary.wiley.com/doi/10.1002/cl2.1135

PROTOCOL: Water, sanitation and hygiene for reducing childhood mortality in low‐ and middle‐income countries

Hugh Sharma Waddington, Sandy Cairncross

## 15 Social Welfare

The effects of creative arts therapy on the treatment of problem users of alcohol and other drugs: A mixed methods systematic review

Arianne Reis, Kaniz Fatema, Philip Hayward, Anna Kearns, Nicole Peel, Gilbert Whitton

https://onlinelibrary.wiley.com/doi/10.1002/cl2.1056

PROTOCOL: Psychosocial interventions for preventing PTSD in children exposed to war and conflict‐related violence: A systematic review

Jennifer Hanratty, Laura Neeson, Tania Bosqui, Michael Duffy, Laura Dunne, Paul Connolly

https://onlinelibrary.wiley.com/doi/10.1002/cl2.1259

PROTOCOL: Effects of virtual reality exposure therapy versus in vivo exposure in treating social anxiety disorder in adults: A systematic review and meta‐analysis

Martin Polak, Norbert Tanzer, Per Carlbring

https://onlinelibrary.wiley.com/doi/10.1002/CL2.109

PROTOCOL: The therapeutic alliance and psychotherapy outcomes for young adults aged 18 to 34

Stacy J. Green, Julia H. Littell, Karianne Hammerstrøm, Emily Tanner‐Smith, Jessica Schaffner Wilen

https://onlinelibrary.wiley.com/doi/10.1002/CL2.56

PROTOCOL: Cognitive‐behavioural therapy for parents who have physically abused their children

Mogens N. Christoffersen, Jacqueline Corcoran, Diane DePanfilis, Claire Daining

